# Relationships between undergraduate medical students’ attitudes toward communication skills learning and demographics in Zambia: a survey-based descriptive study

**DOI:** 10.3352/jeehp.2023.20.16

**Published:** 2023-06-01

**Authors:** Mercy Ijeoma Okwudili Ezeala, John Volk

**Affiliations:** 1 Departmet of Psychiatry, School of Medicine, University of Zambia, Lusaka, Zambia; 2 School of Postgraduate Studies, Africa Research University, Lusaka, Zambia; Hallym University, Korea

**Keywords:** Attitude, Communication, Student, Undergraduate medical education, Zambia

## Abstract

**Purpose:**

This study aimed to detect relationships between undergraduate students’ attitudes toward communication skills learning and demographic variables (such as age, academic year, and gender). Understanding these relationships could provide information for communication skills facilitators and curriculum planners on structuring course delivery and integrating communication skills training into the medical curriculum.

**Methods:**

The descriptive study involved a survey of 369 undergraduate students from 2 medical schools in Zambia who participated in communication skills training stratified by academic year using the Communication Skills Attitude Scale. Data were collected between October and December 2021 and analyzed using IBM SPSS for Windows version 28.0.

**Results:**

One-way analysis of variance revealed a significant difference in attitude between at least 5 academic years. There was a significant difference in attitudes between the 2nd and 5th academic years (t=5.95, P˂0.001). No significant difference in attitudes existed among the academic years on the negative subscale; the 2nd and 3rd (t=3.82, P=0.004), 4th (t=3.61, P=0.011), 5th (t=8.36, P˂0.001), and 6th (t=4.20, P=0.001) academic years showed significant differences on the positive subscale. Age showed no correlation with attitudes. There was a more favorable attitude to learning communication skills among the women participants than among the men participants (P=0.006).

**Conclusion:**

Despite positive general attitudes toward learning communication skills, the difference in attitude between the genders, academic years 2 and 5, and the subsequent classes suggest a re-evaluation of the curriculum and teaching methods to facilitate appropriate course structure according to the academic years and a learning process that addressees gender differences.

## Graphical abstract

[Fig f3-jeehp-20-16]

## Introduction

### Background

An approach to medical training that provides students with the expertise, professional attitudes, and values to encourage quality practice and clinical competence is vital for responding to the changing milieu of the healthcare system and the expected culture of accountability and quality services required by society. This approach integrates communication and interpersonal skills with clinical knowledge and skills to respond appropriately to patients, healthcare teams, and the community. Communication benefits the patient, healthcare, and the economic system [1]. It consists of skills that students can learn. Communication skills are central to enhancing students’ competence in interpersonal relationships, self-assurance, and clinical practice. Hardee et al. [2] reported that intensive communication skills training increased patients’ satisfaction with medical personnel, and the effects of the training lasted even 12 months after training. Students’ attitudes, habits, and environmental factors contribute to the meaning they construe from their learning experiences. Therefore, demographic parameters and the learning environment could influence students’ attitudes toward learning communication skills and may predict the success of training [3].

An attitude reflects a favorable or non-favorable response to a phenomenon, usually expressed through behavior and arising from experience. A student often exhibits behaviors that align with their perception of the learning environment [4], and a doctor’s attitude may affect the quality of communication with a client. Studies have posited that methods of learning communication skills, year of study, demographic details such as age, gender, parents’ occupation, and learning experiences affect medical students’ responses or attitudes to variables such as the time they are willing to invest in studying and practicing skills [5,6]. Medical students’ attitudes toward communication skills may decline as they progress in their studies [7]. Furthermore, their sociocultural background may influence their perception of communication skills [8]. This brief review indicates that medical students’ attitudes to various variables related to communication skills are vital for acquiring skills needed for effective physician-client relationships.

### Objectives

This study endeavored to identify whether an association existed between medical students’ attitudes to communication skills learning and demographic variables such as age, gender, and academic year, with the intent of highlighting the importance of addressing individual differences during communication skills training for an effective outcome.

## Methods

### Ethics statement

The researchers obtained ethical clearance from the Mulungushi University School of Medicine and Health Sciences Ethical Review Committee (reference number: SMHS-MU3-2021-17) and study permission from the National Health Research Authority (reference number: NHRA00014/29/09/2021). Written permission from the deans of the 2 medical schools that participated in the study to survey their students was obtained before data collection. The participants endorsed the consent forms after understanding the study objectives and expectations from them.

### Study design

This was a survey-based, descriptive study. It was described according to the STROBE statement (https://www.strobe-statement.org).

### Settings

The study collected data from participants at the School of Medicine and Health Sciences, Mulungushi University, Livingstone Campus, and the School of Medicine, University of Zambia, Ridgeway Campus, Lusaka, Zambia, between October and December 2021. Mulungushi University (MU) provided one-off communication skills training during the first semester to second-year undergraduate medical students in combination with psychology. The University of Zambia (UNZA) combined communication skills with ethics and professionalism, which it offered for an academic session to 5th-year medical students. Acceptance into medical schools in Zambia often occurs after the 1st or 2nd year of undergraduate studies in natural science courses. MU admitted students into the medical school in the 2nd year and UNZA in the 3rd year during the study period. The 2 medical schools operated similar competency-based curricula integrated with community-based practice, despite the contrast in communication skills’ course timing and length. Both schools had similar communication skills content (Supplement 1).

### Participants

The participants were randomly selected from 315 undergraduate medical students from MU and 446 from UNZA that registered for and participated in communication skills training using an online randomizer (available at: https://www.randomizer.org/). To ensure representation of all the population units and assure reliable responses to questions about relationships, the 256 randomly selected students were stratified according to their academic years. Data collection involved academic years 2 to 7 from the undergraduate medical programs of the participating institutions.

### Variables

The variables included the 26-item questionnaire and its positive and negative subscales. Each subscale contains 13 items.

### Data sources/measurements

The Communication Skills Attitude Scale (CSAS) developed by Rees et al. [9] to investigate medical students’ attitudes regarding communication skills facilitated this study’s data collection (Supplement 2). Each of the 26 items of the CSAS has a 5-point Likert-style scale, ranging from strongly disagree (represented as 1) to strongly agree (corresponding to a score of 5). The developers structured 13 items as positive statements about the importance of communication skills in relationship building and medical training; the 13 other statements constitute the negative attitude subscale, highlighting the worthlessness of studying communication skills in the medical program. The maximum score for the 26 items is 130, and each subscale has 65 as its maximum. Higher scores indicate a more positive attitude towards communication skills learning. John Wiley and Sons permitted this study to use the CSAS. Dataset 1 is the response data.

### Bias

The study used class representatives as research assistants during data collection to minimize any effect of the power differential.

### Study size

This study purposively selected MU and UNZA out of the 8 medical schools in Zambia at the time of this study. Open-source Epidemiologic Statistics for Public Health version 3 (OpenEpi, available at: https://www.openepi.com), facilitated the computation of the sample size based on a 95% confidence level, 5% margin of error, 50% hypothesized response distribution, design effect, and population size, resulting in a sample size of 256 out of the 761 elements. In total, 369 of the 385 questionnaires distributed were suitable for analysis, increasing the confidence level to more than 99%, thereby reducing the 5% margin of error. The response rate was 96.1%.

### Statistical methods

**Fig. 1.**
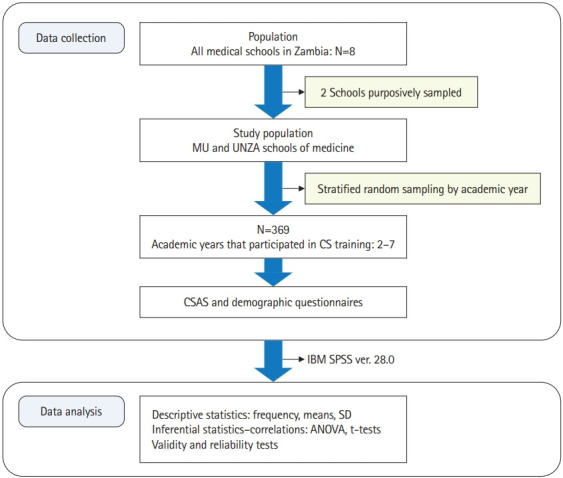
Flow chart for current study protocol summarizing the data collection and analysis process. MU, Mulungushi University; UNZA, University of Zambia; CS, communication skills; CSAS, Communication Skills Attitude Scale; SD, standard deviation; ANOVA, analysis of variance.

The data were anonymized and the negative item scores were reverse-coded before entering them into IBM SPSS ver. 28.0 (IBM Corp.) for statistical analysis. Statistical analysis included the study participants’ global CSAS scores based on the academic year using percentages, numbers, means, and standard deviations. Furthermore, analysis of variance (ANOVA), the independent t-test, and Pearson correlation were used to determine the relationships of academic year, gender, and age with attitudes. Post hoc tests using Tukey’s honestly significant difference verified the equality of variance. The Levene, Bartlett, and Kaiser-Meyer-Olkin tests were conducted to ascertain the fitness of the dataset for analysis. Additionally, the reliability of the CSAS was tested using the Cronbach α, and confirmatory factor analysis was conducted for validity. Fig. 1 summarizes the study protocol.

## Results

### Participants

Participants’ demographic characteristics are shown in Table 1.

### Main results

**Fig. 2.**
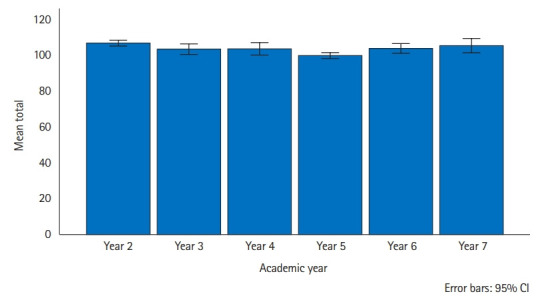
Distribution of the Communication Skills Attitude Scale scores of medical students in Zambia by the academic year. CI, confidence interval.

The results of the CSAS for the global and subscale scores showed more positive than negative attitudes toward communication skills learning among the 369 study participants. The mean scores of the total, positive attitude scale (PAS), and negative attitude scale (NAS) were 103.6 (78%±9.5%), 55.5 (85%±5.2%), and 48.0 (74%±5.8%), respectively. Fig. 2 shows the distribution of the CSAS scores among academic years with a 95.0% confidence interval. Year 2 had a mean score of 107.0 (82%±9.2%), year 3 was 103.6 (80%±7.9%), and year 4 scored 103.8 (80%±7.6%). Year 5 had a mean score of 100.0 (77%±9.7%), year 6 scored 104 (80%±8.6%), and year 7 had a mean score of 105.6 (81%±7.5%).

The subscales similarly recorded high scores, indicating favorable attitudes toward learning communication skills (Table 2). Year 5 had lower scores than the other academic years in both subscales.

The test of homogeneity of variance among the academic years for the 26 CSAS items suggested no significant difference (P=0.689), indicating equal variability among the academic years. One-way ANOVA was used to compare students’ attitudes toward communication skills learning in different academic years. The results revealed a statistically significant difference in attitudes between at least 5 academic years (F [5, 363]=[7.880], P=0.001) (Table 3). The Tukey post hoc test to determine whether any significant difference existed in attitudes among the academic years on the global CSAS showed a significant difference between years 2 and 5 (t=5.95, P˂0.001) (Supplement 3). On the subscales, the results suggested homogeneity of variance (PAS: P=0.379; NAS: P=0.476). There were no significant differences in the mean NAS scores of the different academic years, unlike the positive attitude results. The PAS results indicated significant differences in attitudes between years 2 and 3 (t=3.82, P=0.004), 2 and 4 (t=3.61, P=0.011), 2 and 5 (t=8.36, P˂0.001), and 2 and 6 (t=4.20, P=0.001) (Supplement 4). Year 7 did not have any significant differences in attitude from the other academic years. However, a statistically significant difference was found between years 2 and 5.

The t-test for the difference in means between the men and women participants showed that the women participants had a more positive attitude toward learning communication skills than the men (t [367]=-2.783, P=0.006) (Supplement 5). Age did not correlate with the CSAS total score (R^2^=0.001, P=0.481).

Exploratory factor analysis yielded 8 factors with eigenvalues less than 1 accounting for 57.6% of the total variance (Supplement 6); the original scale yielded 6 factors that accounted for 56.7% of the variances [9]. Confirmatory factor analysis for the construct validity of the CSAS showed that the original 2 factors accounted for 30.4% of the total variance. A scree plot confirmed 2 predominant factors in this study (Supplement 7). A measure of the sample’s suitability yielded a value of 0.869 (Supplement 8). The test of sphericity with a result of P<0.001 indicated the adequacy of the dataset for the study. The original scale had a Cronbach α of 0.87 for the PAS and 0.80 for the NAS [9]. This study’s Cronbach α was 0.80 for all 26 items, 0.73 for the PAS, and 0.70 for the NAS (Supplement 9). An internal consistency of 0.60 is considered acceptable [10].

## Discussion

### Key results

The study aimed to determine the relationship between participants’ attitudes toward learning communication skills and factors including academic years, gender, and age. The CSAS global and subscale scores demonstrated favorable attitudes toward communication skills learning in all the academic years. Year 2 participants had a significantly more positive attitude toward their communication skills learning experiences than year 5 participants. Among the 2nd to 6th academic years, year 2 exhibited a more favorable attitude on the PAS. The attitudes of the women participants were more favorable toward communication skills learning than those of the men. The validity and reliability of the CSAS on the study participants demonstrated an acceptable fit.

### Interpretation

This study’s primary objective was to ascertain the factors that affect undergraduate medical students’ attitudes toward learning communication skills. The results demonstrated that despite the positive attitudes toward learning communication skills among the academic years, the students newly admitted into the medical school exhibited more positive attitudes toward learning communication skills, with a higher PAS than those in clinical classes and those who had been in medical school for more than a year. Carpenter et al. [11] suggested that thoughts about the realities of medical practice are often negatively stressful to students in their higher levels of study. Workload and the stress of encountering and interacting with actual patients could have caused the decrease in PAS among the higher and clinical classes, unlike the year 2 students who still operated under the euphoria of their acceptance into medical schools. The year 5 students had to battle with combining communication skills classes with their clinical postings. The marked difference in attitude between years 2 and 5 confirms this view, as the 2nd and 5th academic years demonstrated smaller error bars (Fig. 1), indicating a greater likelihood of CSAS mean representativeness than in the other academic years. Year 7 did not show a significant difference in the PAS, probably because they had gained enough confidence in their communicative ability and interpersonal skills not to be discouraged by the vicissitudes of academic and clinical experiences. Course timing and a stressful learning environment could induce an increasingly unfavorable attitude despite positive experiences with communication skills learning.

The association between female gender and positive attitudes to communication, empathy, and patient-centeredness could be due to the long-lasting stereotypical classification of women as mainly caretakers and linguistically verbose [12]; in contrast, task-oriented men could perform their duties as physicians despite limitations in their communicative abilities. Such stereotypes would continue to limit healthcare standards if not addressed during medical training. The ages were distributed across the levels, since some participants in the lower classes did not start their undergraduate training immediately after high school; this explains the insignificant correlation results between age and attitudes. The different age ranges in each academic year suggest that communication skills teachers should consider versatile learning activities to enhance learning among all students.

### Comparison with previous studies

Other studies also identified an increase in the NAS and a decrease in the PAS as academic years increased [13,14]. Voltmer et al. [13] similarly suggested that favorable attitudes toward communication skills learning only occurred when students eased into their training. Some others observed that positive attitudes increased with academic years [6]. Contrarily, Nourein et al. [6] did not observe gender and age differences in attitudes toward communication skills learning, unlike other studies [14,15].

### Limitations

This study’s main limitation is its cross-sectional design. A pre- and post-test study design would have accounted better for the relationship between the demographic variables and attitudes toward communication skills training.

### Generalizability

This study’s results contribute to the literature on communication skills in undergraduate medical education; they validate and, to a great extent, expand the current understanding of the factors associated with training medical students in communication skills globally, particularly in Southern Africa.

### Suggestions

Future studies should consider a longitudinal study design.

### Conclusion

The global and subscale CSAS scores demonstrated that undergraduate medical students favored communication skills learning in Zambia. The effects of academic years on attitude imply that undergraduate medical education curriculum planners should consider how and when to integrate communication skills to enhance competency in communication skills. Communication skills educators must consider teaching methods that involve and address the learning needs of all students. Such a learning process will facilitate interest in and the development of positive attitudes toward communication skills.

## Figures and Tables

**Figure f3-jeehp-20-16:**
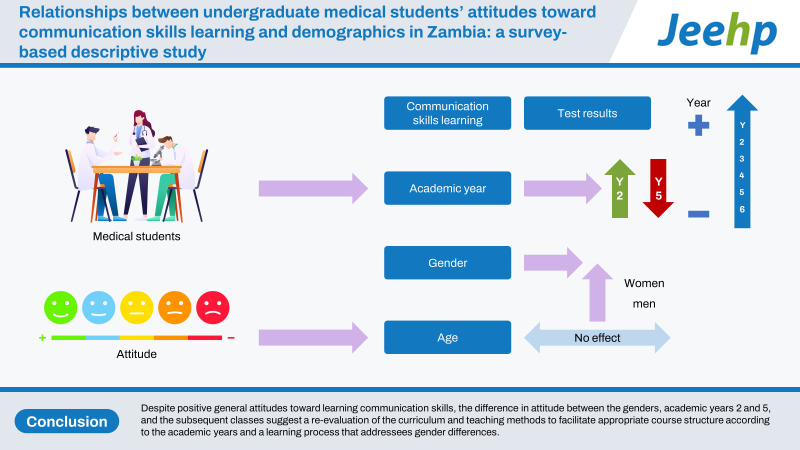


**Table 1.** Demographic characteristics of study participants—medical students in the Mulungushi University School of Medicine and Health Sciences (N=369)

Characteristic	Value
Academic year	
Year 2	125 (34.0)
Year 3	30 (8.0)
Year 4	21 (6.0)
Year 5	136 (37.0)
Year 6	41 (11.0)
Year 7	16 (4.0)
Gender	
Men	212 (57.0)
Women	157 (43.0)
Age (yr)	24.0±3.8 (18–44)

Values are presented as number (%) or mean±standard deviation (range).

**Table 2.** Communication Skills Attitude Scale mean scores of medical students in Zambia by academic year

Academic year	Mean score (%)	
	Positive attitude subscale	Negative attitude subscale
Year 2	58.41(87.0)	48.57 (75.0)
Year 3	54.87 (84.0)	48.70 (75.0)
Year 4	54.62 (84.0)	49.72 (76.0)
Year 5	53.29 (82.0)	46.72 (72.0)
Year 6	55.02 (85.0)	48.95 (75.0)
Year 7	54.88 (84.0)	50.69 (78.0)

**Table 3.** Comparison of students’ attitudes toward communication skills learning in different academic years in medical students in Zambia

	Sum of squares	Degrees of freedom	Mean square	F	Significance
Between groups	3,240.123	5	648.025	7.880	<0.001
Within groups	29,852.988	363	82.240		
Total	33,093.111	368			

## Footnotes

**Data availability**
Data files are available from Harvard Dataverse: https://doi.org/10.7910/DVN/CEOWED Dataset 1. The data file contains the communication skills attitude scale response data.

## Supplementary materials

Supplementary files are available from Harvard Dataverse: https://doi.org/10.7910/DVN/CEOWED

Supplement 1. Communication skills curricula of the 2 participating medical schools.

Supplement 2. The Communication Skills Attitude Scale.

Supplement 3. Multiple comparisons of mean scores by academic years.

Supplement 4. Multiple comparisons of positive attitude scale mean scores by academic years.

Supplement 5. Results of the independent sample t-test for gender.

Supplement 6. Loading of the principal factors of the Communication Skills Attitude Scale.

Supplement 7. Scree plot of the principal component analysis.

Supplement 8. KMO and Bartlett test results for the dataset.

Supplement 9. Reliability test results.

Supplement 10. Audio recording of the abstract.
