# Supplementary material for: Relationships between undergraduate medical students’ attitudes toward communication skills learning and demographics in Zambia: a survey-based descriptive study
Source: J Educ Eval Health Prof. 2023 Jun 1;20:16. doi: 10.3352/jeehp.2023.20.16 (PMC10315251; doi:10.3352/jeehp.2023.20.16)
Supplement: Supplementary file 2 — Supplement 1. Communication skills curricula of the 2 participating medical schools. [file jeehp-20-16-suppl1.docx]

**Supplement 1.** Communication skills curricula of the participating medical schools

**A. Communication skills curriculum of Mulungushi University school of medicine**

**Course title: Behavioral sciences**

**Course code: MBC271**

**Part 2. Communication skills & professionalism**

**1. Rationale**

The relationship between health professionals, the patients, and society is central to encouraging healthy behaviors. Several factors including communication affect this relationship. Poor communication and unprofessional behaviors can lead to a negative patient-provider-society relationships resulting in poor adherence to treatment; extended stay in hospitals, extensive health cost, and has adverse effects on health practitioner’s time management, collaborative relationships, and expertise. Effective communication is at the heart of patient care. It encourages accurate identification of clients’ problems based on facts collected from the clients’ expressed needs rather than on assumptions. The communication skills and professionalism aspect of MBC271 course enables the students to develop academic writing, research, presentation, listening and study skills, improve their nonverbal, verbal and paralanguage communication behavior, thereby enhancing their competence as medical professionals. The course also introduces the students to cultural and ethical issues in healthcare relating to communication.

**2. Aim**

To provide the students with the knowledge, skills and attitudes that enhance effective communication, professionalism and personal academic development required for effective participation in the health sector and the academic/scientific community.

**3. Objectives**

At the end of this course, the student should be able to:

1) Explain and evaluate the concepts and factors that affect communication

2) Demonstrate appropriate interview, listening and presentation skills

3) Evaluate practical communication and patient-centered skills necessary for attending to clients, colleagues, and society

4) Demonstrate academic research, writing and documentation skills

5) Utilize appropriate study and ICT (information and communications technology) skills

6) Illustrate collaborative and interpersonal skills centered on empathy, respect, and trust

7) Evaluate the ethical issues in doctor patient relationship

8) Demonstrate sensitivity to ethical and cultural issues in communication

9) Apply patient-centered communication during consultation, sharing health-related, and lifestyle information.

10) Elucidate the roles, rules and status that govern communication in a health care setting

**4. Learning outcomes**

The graduate should be able to:

1) Listen actively and critically to information communicated by colleagues and clients from the speaker’s perspective

a. Observes, interprets, and evaluates the verbal, nonverbal and paralanguage communication of patients and others, and the nuances of information being transmitted

b. Conducts medical interviews and collects information based on ethical principles

c. Documents correct and factual information in a concise and lucid manner

d. Develops enhanced diagnostic skills based on the explicit and implicit communication behaviors of client

e. Communicates information about health needs, intervention options and their effects clearly at the client’s level of understanding, minimizing the use of medical jargon

2) Communicate empathetically with patients

a. Collects and documents information from the client’s perspective

b. Individualizes every doctor-patient encounter

c. Applies a patient-centered communication approach

3) Communicate factual information to the patients, colleagues and society concisely and lucidly

a. Verbally communicates information in a legible and lucid manner

b. Applies appropriate nonverbal cues to enhance the verbal

c. Demonstrates the professional rules guiding communication in the healthcare field

d. Illustrate appropriate research skills by acknowledging the resources used in writing or presentations

4) Relate collaboratively with other health workers and inter professional members for the patient’s benefit

a. Understands and applies the rules governing every communication situations with people from different status/roles

b. Relates respectfully and positively with medical colleagues and people from other professions/inter professional competence

c. Communicates effectively through verbal and nonverbal means to achieving the team’s goals—offering the most cost effective and patient-centered care to clients

5) Have acquired the skills required to communicate effectively with different groups of people

a. Understands the roles expectations

b. Demonstrates understanding of the effects of time, setting, attitudes and values on health-related information

c. Establishes rapport with people from different age and cultural groups

d. Applies communication aids for clarity and reinforcement, when needed

e. Provides effective feedbacks to clients

6) Apply the principles of medical ethics in decision making and communicating decisions

a. Engages client in full disclosure of medical details including diagnosis, intervention options at the client’s level of understanding

b. Listens and notes every point of view

c. Integrates client/family/colleagues points of view and analyzes them based on ethical principles

d. Expects and respects differing opinions

e. Develops the capacity to justify own opinion reasonably

f. Applies fairness to every client regardless of income, culture, education, age, status, being altruistic

g. Applies honesty and integrity in interacting with clients, their families, professional colleagues, and society/organizations

**5. Course content**

**Unit 1. Introduction: study and basic research skills**

1) Referencing-Harvard style and plagiarism

2) Planning work and organizing resources

3) Time management skills

4) Reflective practices

5) Reading skills: the reading process and strategies

6) Examination skills and exam malpractice

7) Team building skills

8) Library use and resources

**Unit 2. Principles of communication**

1) Communication models, goals, and levels

2) Social situations governing communication (roles, rules and status and their effect in the health sector)

3) Forms of communication: verbal, non-verbal paralanguage, and alternative forms

**Unit 3. Communication barriers**

1) External/physical communication barriers

2) Interpersonal communication barriers

3) Intrapersonal communication barriers

4) Semantic/language barriers

5) Physiological barriers/health issues

6) Organizational barriers

7) Communication barriers and their effects on the health sector

8) Minimizing/overcoming communication barriers

**Unit 4. Factors affecting communication**

1) Communication behavioral styles (assertive, aggressive and submissive/passive)

2) Doctor related factors (physical and psychological factors, training in communication skills and field expertise)

3) Patient-related factors (physical and psychological factors, previous and current experiences)

4) Physical setting

5) Attribution and perception process

**Unit 5. Effective communication and collaborative healthcare**

1) Active/effective listening

a. The listening purposes and process

b. Types of listening: discriminative, informational, critical, and empathetic

c. Styles of listening

d. Barriers to listening and strategies for overcoming the barriers

2) Use of appropriate nonverbal cues

3) Tone, pace, and inflections

4) Doctor-patient relationships: role expectations

5) Doctor-patient communication relationship styles (paternalistic approach, patient-centered approach, mutuality approach and default style)

6) Characteristics of collaborative health care and power relations

7) Barriers to collaborative environment

8) Therapeutic communication

**Unit 6. Gender, sexuality, and culture**

1) Gender and cultural differences in communication

2) Communicating with a patient of a different gender and/or sex

3) Communicating with a patient from a different culture

4) Effects of gender/culture/sexuality on health professional’s practice

5) Models for intercultural communication

**Unit 7. History taking and interview skills**

1) Importance and characteristics of medical interviews

2) Functions of the medical interviews: diagnostic and therapeutic

3) Models and steps of the medical interviews

4) Barriers associated with the interviews

**Unit 8. Difficult situations and breaking bad news**

1) Types and characteristics of difficult patient/situations and their communication

2) Approaches to difficult encounters/patients

3) Definition of and reasons for breaking bad news

4) Models for breaking bad news: SPIKES, BATHE, etc.

**Unit 9. Communicating with vulnerable patients**

1) Characteristics

2) Communicating with the family of the vulnerable

3) Communicating with children and adolescents

4) Communicating with the aged/elderly client

a. Age-related changes affecting communication

b. Expressing concern

5) Expected sources of communication barrier and strategies for minimizing them

**Unit 10. Academic writing**

1) Principles of writing

2) Structures, organization and writing styles

3) Types of writing:

a. Essays and summary writing: paraphrasing

b. Formal/business writing: reports, memos, meeting minutes, resumes, and cover letters

**Unit 11. Giving information, documentation, and oral presentation**

1) Guidelines on giving information to patient

2) Giving information to a group: reports, case conferences and discharge meetings, meeting with relatives, therapeutic/activity groups

3) Giving lifestyle advice

4) Use of written information

5) Obtaining informed consent

6) Definition of oral presentation

7) Preparation for presentations

8) Presentation structures

9) Delivery of presentations

10) Visual aids

**Unit 12. Electronic communication and the internet**

1) Use of the internet for health-related information and support

2) Health information communicated through the internet

3) Effects of the internet on patient-physician relationships

4) Documenting and transferring patient’s records electronically and privacy

5) Academic integrity and the use of the internet (plagiarism and using internet resources-revisited)

**Unit 13. Professionalism**

1) History and characteristics: right and good healing

2) Values and behaviors expected of the medical professional

3) Challenges in teaching-learning medical professionalism

**6. Teaching methods: 2 contact hours/week including**:

1) Lectures

2) Seminars

3) Small group discussions

4) Project (evidence-based): student presentations

5) Practical role plays, problem solving, decision making

6) Case studies

7) Audio-visual feedback

8) Simulations

**7. Assessment methods**

1) Written

2) Presentations

3) Projects

(1). Continuous assessment: 40%

a. Tests

b. Projects: essays/letters/reports

c. Practical/seminar/oral presentation

(2) Exam: 60%

a. MCQ

b. Case scenarios

c. Short answers

d. Picture interpretations

**8. Prescribed textbooks**

1. Byyny, R.L., Papadakis, M.A., & Paauw, D.S. (2015). Medical professionalism best practices. California: Alpha Omega Alpha Honor Medical Society Menlo Park.

2. Lloyd, M, Bor, R and Noble, N. (2018). Clinical communication skills for medicine. Elsevier (eBook).

3. Kissane, et al (Eds.) (2017). Oxford textbook of communication and palliative care. Oxford: Oxford University Press.

4. Silverman, J., Kurtz, S and Draper, J. (2013). Skills for communicating with patients. NY: Taylor and Francis.

5. Serveilles, v. G. (2009). Communication skills for the health care professionals. London: Jones and Bartlett publishers.

**9. Recommended textbooks**

1. Tate, P and Frame, F. (2018). The doctor communication handbook. FL: Taylor and Francis.

2. Lloyd, M (2009). Communication skills for medical practice. Churchill Livingstone. Clinical Communication Skills for Medicine.

3. Tamparo, C.D and Lindh, W.Q. (2013). Therapeutic communications for healthcare. Delmar Cengage Learning.

4. Smith, R.C. 2002. Patient-centred interviewing: an evidence-based method. Philadelphia: Lippincott Williams & Wilkins.

5. Faulkner, A. (2004). Effective interaction with patients. Livingstone: Churchill.

6. Macdonald, E. (ed.) (2004). Difficult conversations in medicine. Oxford: Oxford University Press.

7. Davies, P. et al (Eds.) (2017). Medical ethics, law and communication at a glance. West Sussex: Wiley Blackwell.

8. Communicating with patients: a quick reference guide for clinicians.

**B. Communication skills curriculum of the University of Zambia’s school of medicine**

**Course title: communication skills, professionalism & health care ethics**

**Course code: PSY5215**

**Part 1. Communication skills and professionalism**

**1. Rationale**

The relationship between the health workers and patients plays an important role in encouraging healthy behaviors. Several factors including communication affect this relationship. Poor communication can lead to a more negative patient-provider relationship resulting in poor adherence to treatment; extended stay in hospitals, extensive health cost, and impacts poorly on health practitioner’s time management, collaborative relationships, and expertise. Effective communication is at the heart of patient care. It encourages accurate identification of clients’ problems based on facts collected from the clients’ expressed needs rather than on assumptions. Psy5215 enables the students to improve on their nonverbal, verbal and paralanguage aspects of communication, thereby enhancing their competence as medical practitioners. The course also introduces the students to cultural and ethical issues in healthcare relating to communication.

**2. Aim**

To provide the students with an understanding of the factors that influence effective communication and to promote sensitivity to ethical and cultural issues in communication.

**3. Objectives**

At the end of this course, the student should be able to:

1) Understand the centrality of good communication to safe patient care

2) Evaluate the factors affecting communication

3) Identify barriers to effective communication especially within the health care system and suggest solutions to the identified barriers

4) Explain the constituents of effective and active listening

5) Demonstrate appropriate interview skills

6) Evaluate practical communication skills required for physically attending to clients

7) Identify and manage the language needs and communication preferences of clients and their relations

8) Identify when and how to use the different types of questions in a health care setting

9) Prepare and conduct oral presentations tailored to meet an audience’s need

10) Elucidate the roles, rules and status that govern communication in a health care setting

11) Identify what constitutes bad news and the principles of breaking bad news

12) Acquire the skills required to communicate effectively with different groups of people

13) Explain the concept of therapeutic communication

14) Appraise the use of technology in communication within the health care setting

15) Evaluate the ethical issues in the doctor-patient relationship

16) Explain the relationship between gender, culture, and communication

17) Assertively yet professionally communicate concerns about patients that are not being properly addressed

18) Demonstrate appropriate writing and documentation style

**4. Learning outcomes**

The graduates should be able to:

1) Listen actively to information communicated by colleagues and clients

a. Observes and interprets the verbal, nonverbal and paralanguage communication of patients and others, and the nuances of information being transmitted

b. Conducts medical interviews and collects information based on ethical principles

c. Documents correct and factual information in a concise and lucid manner

d. Develops enhanced diagnostic skills based on the explicit and implicit communication behaviors of client

e. Communicates information about health needs, intervention options and their effects clearly at the client’s level of understanding, minimizing the use of medical jargon

2) Communicate empathetically with patients

a. Collects and documents information from the client’s perspective

b. Individualizes every doctor-patient encounter

c. Applies a patient-centered communication approach

3) Communicate factual information to the patients, colleagues and society concisely and lucidly

a. Verbally and/or orally communicate information in a legible and lucid manner

b. Demonstrates the professional rules guiding communication in the healthcare field

c. Acknowledges the resources used in one’s writing

4) Relate collaboratively with other health workers and inter professional members for the patient’s benefit

a. Understands and applies the rules governing every communication situations with people from different status/roles

b. Relates respectfully with medical colleagues and people from other professions

c. Responds positively to help from other professions/inter professional competence

d. Communicates effectively through verbal and nonverbal means to achieving the team’s goals—offering the most cost effective and patient-centered care to clients

5) Have acquired the skills required to communicate effectively with different groups of people

a. Understands the roles expectations

b. Demonstrates understanding of the effects of time, setting, attitudes and values on health-related information

c. Establishes rapport with people from different age and cultural groups

d. Applies communication aids for clarity and reinforcement, when required

e. Provides effective feedback to clients

**5. Course content**

**Unit 1. Introduction**

1) Basic principles of communication skills (Communication models, goals, and levels)

2) Social situations governing communication (roles, rules, and status and their effect in communication within the health sector)

3) Forms of communication (verbal, nonverbal, and paralanguage)

4) Alternative forms of communication

**Unit 2. Communication barriers**

1) External/physical communication barriers

2) Interpersonal communication barriers

3) Intrapersonal communication barriers

4) Semantic/language barriers

5) Physiological barriers/health issues

6) Organizational barriers

7) Communication barriers and their effects on the health sector

8) Minimizing/overcoming communication barriers

**Unit 3. Factors affecting communication**

1) Communication behavioral styles (assertive, aggressive, and submissive/passive)

2) Doctor related factors (physical and psychological factors, training in communication skills and field expertise

3) Patient-related factors (physical and psychological factors, previous and current experiences)

4) Physical setting

**Unit 4. Effective communication and collaborative health care**

1) Active/effective listening

2) Use of appropriate nonverbal cues

3) Tone, pace, and inflections

4) Doctor-patient relationships: role expectations

5) Doctor-patient communication relationship styles (paternalistic approach, patient-centered approach, mutuality approach, and default style)

6) Characteristics of collaborative health care

7) Barriers to collaborative environment

8) Therapeutic communication

**Unit 5. Use of questions**

1) Therapeutic use of questions

2) Non therapeutic use of questions

3) Types of questions

4) Choice of questions

**Unit 6. Silence and pauses**

1) Definition

2) Functions/uses of silence

3) Interpretation of silence in patient’s response

4) Negative use of silence

**Unit 7. Gender and culture**

1) Gender and cultural differences in communication

2) Communicating with a patient of a different gender and/or sex

3) Communicating with a patient from a different culture

4) Effects of gender/culture on health professional’s practice

**Unit 9. History taking**

1) Importance of medical interviews

2) Characteristics

3) Diagnostic functions of the interview

4) Therapeutic functions of the interview

5) Models of history taking

6) Steps of the medical interviews

7) Barriers associated with the interviews

**Unit 10. Breaking ‘bad’ news**

1) Definitions

2) Reasons for breaking ‘bad’ news

3) Why the difficulty in breaking ‘bad’ news?

4) Who breaks ‘bad’ news, to whom and when?

5) Models for breaking ‘bad’ news: the SPIKES model

**Unit 11. Communicating with difficult patients/situations**

1) Types of difficult patients and their communication

2) Characteristics

3) Approaches to difficult encounters/patients

**Unit 12. Communicating with vulnerable people**

1) Characteristics

2) Communicating with the family of the vulnerable

3) Communicating with children and adolescents

4) Expected sources of communication barrier

5) Communicating with the aged

6) Age-associated changes that affect communication

7) Expressing concern

**Unit 13. Giving information, documenting information, and oral presentations**

1) Guidelines on giving information to patient

2) Giving information to a group: reports, case conferences and discharge meetings, meeting with relatives, therapeutic/activity groups

3) Giving lifestyle advice

4) Use of written information

5) Obtaining informed consent

6) Definition of oral presentation

7) Preparation for presentations

8) Presentation structures

9) Delivery of presentations

10) Visual aids

**Unit 14. Electronic communication and the internet**

1) Use of the internet for health-related information and support

2) Health information communicated through the internet

3) Effects of the internet on patient-physician relationships

4) Documenting and transferring patient’s records electronically and privacy

5) Academic integrity and the use of the internet (plagiarism and using internet resources)

**6. Prescribed textbooks**

1. Lloyd, M, Bor, R and Noble, N. (2018). Clinical communication skills for medicine. Elsevier (eBook).

2. Kissane, et al (Eds.) (2017). Oxford textbook of communication and palliative care. Oxford: Oxford University Press.

3. Silverman, J., Kurtz, S and Draper, J. (2013). Skills for communicating with patients. NY: Taylor and Francis.

4. Serveilles, v. G. (2009). Communication skills for the health care professionals. London: Jones and Bartlett publishers.

**7. Recommended textbooks**

1. Tate, P and Frame, F. (2018). The doctor communication handbook. FL: Taylor and Francis.

2. Lloyd, M (2009). Communication skills for medical practice. Churchill Livingstone. Clinical Communication Skills for Medicine.

3. Tamparo, C.D and Lindh, W.Q. (2013). Therapeutic communications for healthcare. Delmar Cengage Learning.

4. Smith, R.C. 2002. Patient-centred interviewing: an evidence-based method. Philadelphia: Lippincott Williams & Wilkins.

5. Faulkner, A. (2004). Effective interaction with patients. Livingstone: Churchill.

6. Macdonald, E. (ed.) (2004). Difficult conversations in medicine. Oxford: Oxford University Press.

7. Communicating with patients: a quick reference guide for clinicians.

8. Difficult consultations with adolescents & working with young people: evidence-based health communication & communicating health – Manual.
