# Supplementary material for: Relationships between undergraduate medical students’ attitudes toward communication skills learning and demographics in Zambia: a survey-based descriptive study
Source: J Educ Eval Health Prof. 2023 Jun 1;20:16. doi: 10.3352/jeehp.2023.20.16 (PMC10315251; doi:10.3352/jeehp.2023.20.16)
Supplement: Supplementary file 3 — Supplement 2. The Communication Skills Attitude Scale. [file jeehp-20-16-suppl2.docx]

**Supplement 2.** Communication skills attitude scale questionnaire

| Questionnaire statement | SA (5) | Agree (4) | Neutral or unsure (3) | DA (2) | SD (1) |
| --- | --- | --- | --- | --- | --- |
| 1. To be a doctor I must have good CS |  |  |  |  |  |
| 2. I can’t see the point in learning CS |  |  |  |  |  |
| 3. Nobody is going to fail their medical degree for having poor CS |  |  |  |  |  |
| 4. Developing my CS is just as important as developing my knowledge of medicine |  |  |  |  |  |
| 5. Learning CS has helped me or will help me respect patients |  |  |  |  |  |
| 6. I haven’t got time to learn CS |  |  |  |  |  |
| 7. Learning CS is interesting |  |  |  |  |  |
| 8. I can’t be bothered to turn up to sessions in CS |  |  |  |  |  |
| 9. Learning CS helped or will help facilitate my team-working skills |  |  |  |  |  |
| 10. Learning CS has or will improve my ability to communicate with patients |  |  |  |  |  |
| 11. CS teaching states the obvious and then complicates it |  |  |  |  |  |
| 12. Learning CS is fun |  |  |  |  |  |
| 13. Learning CS is easy |  |  |  |  |  |
| 14. Learning CS has helped or will help me respect my colleagues |  |  |  |  |  |
| 15. I find it difficult to trust information about CS given to me by non-clinical lecturers. |  |  |  |  |  |
| 16. Learning CS has helped or will help me recognize patients’ rights regarding confidentiality and informed consent |  |  |  |  |  |
| 17. CS teaching would have a better image if it sounded more like a science subject |  |  |  |  |  |
| 18. When applying for medicine, I thought it was a really good idea to learn CS |  |  |  |  |  |
| 19. I don’t need good CS to be a doctor |  |  |  |  |  |
| 20. I find it hard to admit to having some problems with my CS |  |  |  |  |  |
| 21. I think it’s really useful to learn CS on the medical degree |  |  |  |  |  |
| 22. My ability to pass exams will get me through medical school rather than my ability to communicate |  |  |  |  |  |
| 23. Learning CS is applicable to learning medicine. |  |  |  |  |  |
| 24. I find it difficult to take CS learning seriously. |  |  |  |  |  |
| 25. Learning CS is important because my ability to communicate is a lifelong skill |  |  |  |  |  |
| 26. CS learning should be left to psychology students, not medical students |  |  |  |  |  |

CS, communication skills; SA, strongly agree; DA, disagree; SD, strongly disagree.
