# Supplementary material for: Relationships between undergraduate medical students’ attitudes toward communication skills learning and demographics in Zambia: a survey-based descriptive study
Source: J Educ Eval Health Prof. 2023 Jun 1;20:16. doi: 10.3352/jeehp.2023.20.16 (PMC10315251; doi:10.3352/jeehp.2023.20.16)
Supplement: Supplementary file 4 — Supplement 3. Multiple comparisons of mean scores by academic years. [file jeehp-20-16-suppl3.docx]

**Supplement 3.** Multiple comparisons of mean scores by academic years

| (I) Level | (J) Level | Significance |
| --- | --- | --- |
| **Year 2** | Year 3 | 0.436 |
|  | Year 4 | 0.663 |
|  | Year 5 | **<0.001** |
|  | Year 6 | 0.442 |
|  | Year 7 | 0.992 |
| Year 3 | Year 2 | 0.436 |
|  | Year 4 | 1.000 |
|  | Year 5 | 0.378 |
|  | Year 6 | 1.000 |
|  | Year 7 | 0.981 |
| Year 4 | Year 2 | 0.663 |
|  | Year 3 | 1.000 |
|  | Year 5 | 0.492 |
|  | Year 6 | 1.000 |
|  | Year 7 | 0.991 |
| Year 5 | Year 2 | **<0.001** |
|  | Year 3 | 0.378 |
|  | Year 4 | 0.492 |
|  | Year 6 | 0.142 |
|  | Year 7 | 0.191 |
| Year 6 | Year 2 | 0.442 |
|  | Year 3 | 1.000 |
|  | Year 4 | 1.000 |
|  | Year 5 | 0.142 |
|  | Year 7 | 0.991 |
| Year 7 | Year 2 | 0.992 |
|  | Year 3 | 0.981 |
|  | Year 4 | 0.991 |
|  | Year 5 | 0.191 |
|  | Year 6 | 0.991 |
| Year 7 | Year 2 | 0.981 |
|  | Year 3 | 0.957 |
|  | Year 4 | 0.978 |
|  | Year 5 | 0.116 |
|  | Year 6 | 0.982 |

Statistically significant results are marked in bold.
