# Supplementary material for: Relationships between undergraduate medical students’ attitudes toward communication skills learning and demographics in Zambia: a survey-based descriptive study
Source: J Educ Eval Health Prof. 2023 Jun 1;20:16. doi: 10.3352/jeehp.2023.20.16 (PMC10315251; doi:10.3352/jeehp.2023.20.16)
Supplement: Supplementary file 5 — Supplement 4. Multiple comparisons of positive attitude scale mean scores by academic years. [file jeehp-20-16-suppl4.docx]

**Supplement 4.** Multiple comparisons of the positive attitude scale mean scores by academic years

| (I) Level | (J) Level | Mean difference (I–J) | Standard error | Significance |
| --- | --- | --- | --- | --- |
| Year 2 | Year 3 | **3.541** | 0.974 | **0.004** |
|  | Year 4 | **3.789** | 1.130 | **0.011** |
|  | Year 5 | **5.114** | 0.594 | **<0.001** |
|  | Year 6 | **3.384** | 0.863 | **0.001** |
|  | Year 7 | 3.533 | 1.273 | 0.064 |
| Year 3 | Year 2 | **-3.541** | 0.974 | **0.004** |
|  | Year 4 | 0.248 | 1.364 | 1.000 |
|  | Year 5 | 1.573 | 0.967 | 0.581 |
|  | Year 6 | -0.158 | 1.151 | 1.000 |
|  | Year 7 | -0.008 | 1.484 | 1.000 |
| Year 4 | Year 2 | **-3.789** | 1.130 | **0.011** |
|  | Year 3 | -0.248 | 1.364 | 1.000 |
|  | Year 5 | 1.325 | 1.124 | 0.847 |
|  | Year 6 | -0.405 | 1.286 | 1.000 |
|  | Year 7 | -0.256 | 1.590 | 1.000 |
| Year 5 | Year 2 | **-5.114** | 0.594 | **<0.001** |
|  | Year 3 | -1.573 | 0.967 | 0.581 |
|  | Year 4 | -1.325 | 1.124 | 0.847 |
|  | Year 6 | -1.730 | 0.854 | 0.329 |
|  | Year 7 | -1.581 | 1.267 | 0.813 |
| Year 6 | Year 2 | **-3.384** | 0.863 | **0.001** |
|  | Year 3 | 0.158 | 1.151 | 1.000 |
|  | Year 4 | 0.405 | 1.286 | 1.000 |
|  | Year 5 | 1.730 | .854 | 0.329 |
|  | Year 7 | 0.149 | 1.413 | 1.000 |
| Year 7 | Year 2 | -3.533 | 1.273 | 0.064 |
|  | Year 3 | 0.008 | 1.484 | 1.000 |
|  | Year 4 | 0.256 | 1.590 | 1.000 |
|  | Year 5 | 1.581 | 1.267 | 0.813 |
|  | Year 6 | -0.149 | 1.413 | 1.000 |

Statistically significant results are marked in bold.
