# Supplementary material for: Relationships between undergraduate medical students’ attitudes toward communication skills learning and demographics in Zambia: a survey-based descriptive study
Source: J Educ Eval Health Prof. 2023 Jun 1;20:16. doi: 10.3352/jeehp.2023.20.16 (PMC10315251; doi:10.3352/jeehp.2023.20.16)
Supplement: Supplementary file 6 — Supplement 5. Results of the independent sample t-test for gender. [file jeehp-20-16-suppl5.docx]

**Supplement 5.** Result of the independent sample test for gender

| Total | Significance (P-value) | | Mean difference | Standard error difference |
| --- | --- | --- | --- | --- |
|  | One-sided | Two-sided |  |  |
| Equal variances assumed | 0.003 | 0.006 | -1.672 | 0.601 |
| Equal variances not assumed | 0.003 | 0.006 | -1.672 | 0.599 |
