# Supplementary material for: Relationships between undergraduate medical students’ attitudes toward communication skills learning and demographics in Zambia: a survey-based descriptive study
Source: J Educ Eval Health Prof. 2023 Jun 1;20:16. doi: 10.3352/jeehp.2023.20.16 (PMC10315251; doi:10.3352/jeehp.2023.20.16)
Supplement: Supplementary file 7 — Supplement 6. Loading of the principal factors of the Communication Skills Attitude Scale. [file jeehp-20-16-suppl6.docx]

**Supplement 6.** Loading of the principle factors of the communication skills attitude scale

| CSAS items | Total | % of variance | Cumulative % |
| --- | --- | --- | --- |
| 1 | 6.114 | 23.515 | 23.515 |
| 2 | 1.793 | 6.895 | 30.410 |
| 3 | 1.492 | 5.738 | 36.148 |
| 4 | 1.191 | 4.579 | 40.727 |
| 5 | 1.167 | 4.490 | 45.217 |
| 6 | 1.105 | 4.250 | 49.467 |
| 7 | 1.069 | 4.112 | 53.579 |
| 8 | 1.045 | 4.020 | 57.599 |
| 9 | 0.969 | 3.726 | 61.325 |
| 10 | 0.893 | 3.435 | 64.760 |
| 11 | 0.817 | 3.142 | 67.902 |
| 12 | 0.792 | 3.048 | 70.949 |
| 13 | 0.780 | 3.000 | 73.950 |
| 14 | 0.739 | 2.841 | 76.791 |
| 15 | 0.660 | 2.538 | 79.330 |
| 16 | 0.642 | 2.471 | 81.801 |
| 17 | 0.599 | 2.303 | 84.103 |
| 18 | 0.569 | 2.187 | 86.290 |
| 19 | 0.519 | 1.997 | 88.287 |
| 20 | 0.517 | 1.990 | 90.276 |
| 21 | 0.491 | 1.890 | 92.167 |
| 22 | 0.469 | 1.802 | 93.969 |
| 23 | 0.437 | 1.683 | 95.651 |
| 24 | 0.420 | 1.617 | 97.268 |
| 25 | 0.392 | 1.508 | 98.776 |
| 26 | 0.318 | 1.224 | 100.000 |

CSAS, communication skills attitude scale.
