# Supplementary figures and images for: Relationships between undergraduate medical students’ attitudes toward communication skills learning and demographics in Zambia: a survey-based descriptive study
Source: J Educ Eval Health Prof. 2023 Jun 1;20:16. doi: 10.3352/jeehp.2023.20.16 (PMC10315251; doi:10.3352/jeehp.2023.20.16)

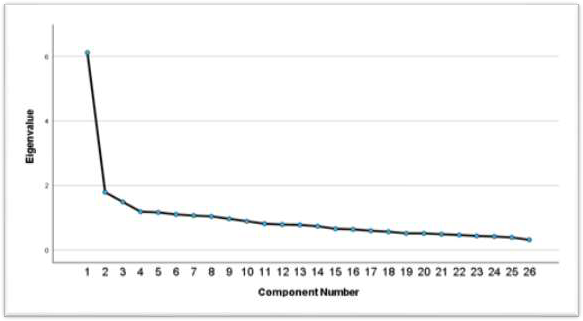


**Supplement 7.** Scree plot of the principal component analysis.

Supplement: Supplementary file 8 — Supplement 7. Scree plot of the principal component analysis. [file jeehp-20-16-suppl7.docx]
