# Supplementary material for: Relationships between undergraduate medical students’ attitudes toward communication skills learning and demographics in Zambia: a survey-based descriptive study
Source: J Educ Eval Health Prof. 2023 Jun 1;20:16. doi: 10.3352/jeehp.2023.20.16 (PMC10315251; doi:10.3352/jeehp.2023.20.16)
Supplement: Supplementary file 9 — Supplement 8. KMO and Bartlett test results for the dataset. [file jeehp-20-16-suppl8.docx]

**Supplement 8.** KMO and Bartlett’s tests results for the dataset

|  | Value |
| --- | --- |
| KMO measure of sampling adequacy | **0.869** |
| Bartlett’s Test of Sphericity |  |
| Approximation chi-square | 2,254.957 |
| Degrees of freedom | 325 |
| Significance | **<0.001** |

Statistically significant results are marked in bold.

KMO, Kaiser-Meyer-Olkin.
