# Supplementary material for: Relationships between undergraduate medical students’ attitudes toward communication skills learning and demographics in Zambia: a survey-based descriptive study
Source: J Educ Eval Health Prof. 2023 Jun 1;20:16. doi: 10.3352/jeehp.2023.20.16 (PMC10315251; doi:10.3352/jeehp.2023.20.16)
Supplement: Supplementary file 10 — Supplement 9. Reliability test results. [file jeehp-20-16-suppl9.docx]

**Supplement 9.** Reliability test results

|  | Cronbach’s α | Cronbach’s α based on standardized items | No. of items |
| --- | --- | --- | --- |
| 26-items CSAS reliability statistics | 0.802 | 0.842 | 26 |
| 13-items positive attitude scale’s reliability statistics | 0.735 | 0.801 | 13 |
| 13-items negative attitude scale’s reliability statistics | 0.699 | 0.712 | 13 |

CSAS, communication skills attitude scale.
